# 

**Published:** 1906-03-24

**Authors:** 


### NOTICE TO CORRESPONDENTS.

All MSS., Letters, Books for Review, and other matters intended for the Editor should be addressed The Editor, "The Hospital," The Hospital Buildings, 28 & 29 Southampton Street, Strand, W.C.

The Editor cannot undertake to return rejected MS., even when accompanied by stamped directed envelope.

**Notice to Advertisers and Subscribers.**

All Advertisements, Orders for Copies of The Hospital, and Business Communications should be addressed to **THE MANAGER (Not to the Editor),** The Hospital, 28 & 29 Southampton Street, Strand London, W.C.

The Cost of Subscription, post free, is as follows:—Per week, 3½d.; per quarter, 3s. 3d.; per half-year, 6s. 6d.; per annum, 13s. Foreign and Colonial:—Per annum, 19s. 6d., payable, in all cases, in advance.

**NOTICE.—Vol. XXXVIII. of "The Hospital" April to September 1905), in handsome cloth binding, will shortly be on sale at the office of this Journal, price 10s. 6d. Cases for binding the Numbers contained in this Volume 2s. Index to Vol. XXXVIII., 3½d., post free.**

**The Monthly Part for March is now ready. Price 1s., by post, 1s. 3d.**

### Medical Appointments and Vacancies.

### VACANCIES.

Bristol General Hospital.—Assistant House Physician.

Bury Infirmary.—Junior House Surgeon.

City of London Hospital for Diseases of the Chest, Victoria Park, E.—Two House Physicians.

East London Hospital for Children and Dispensary for Women, Shadwell, E.—Medical Officer for the Casualty Department.

Farringdon General Dispensary and Lying-in Charity, 17 Bartlett's Buildings, Holborn, E.C.—Honorary Dental Surgeon.

German Hospital, Dalston, N.E.—Honorary Anæsthetist.

Glasgow, Royal Hospital for Sick Children.—Resident House Surgeon. Also Assistant at Dispensary for six months.

Hampstead General Hospital.—Resident Medical Officer.

Hospital for Epilepsy and Paralysis and Other Diseases of the Nervous System, Maida Vale.—Resident Medical Officer.

Italian Hospital.—Honorary Assistant Surgeon.

King's College (University of London).—Sambrooke Medical Registrar.

Manchester Royal Infirmary.—Resident Medical Officer. Also Resident Medical Officer at the Convalescent Hospital, Cheadle.

Margaret Street Hospital for Consumption and Diseases of the Chest, Cavendish Square, W.—Surgeon.

Metropolitan Borough of Battersea.—Appointment of Medical Officer of Health.

National Hospital for the Relief and Cure of the Paralysed and Epileptic, Queen Square, Bloomsbury.—Assistant Surgeon.

Nottingham, Children's Hospital.—House Surgeon.

Queen Charlotte's Lying-in Hospital, Marylebone Road, N.W.—Assistant Resident Medical Officer.

Taunton and Somerset Hospital, Taunton.—Resident Assistant House Surgeon.

Valkenberg Asylum, near Cape Town.—Assistant Medical Officer.

### Books for Midwives and Maternity Nurses

*Just Published.*

**SIMPLE INTRODUCTORY LESSONS IN MIDWIFERY.**

By M. LOANE, Author of "Outlines of Routine in District Nursing." **1/-** net, **1/1** post free

This book is an easy introduction to the theory of Midwifery, for the use of those women who desire ultimately to pass the Central Midwives Board Examination, and who have perhaps some practical experience of midwifery, but who are so entirely ignorant of the theory, and so completely unacquainted with the language in which it is usually set forth, that they are unable to profit immediately by any ordinary courses of lectures on the subject.

**The Best and Latest Handbook on Midwifery.**

**A COMPLETE HANDBOOK OF MIDWIFERY.**

By Dr. J. K. WATSON, Author of "A Handbook for Nurses." **6/-** net, **6/4** post free.

This Handbook has been designed to meet the requirements of the Central Midwives Board. It contains a Complete Course of Midwifery, is very fully illustrated, and is undoubtedly the best Textbook for Nurses entering for the C.M.B. Examinations, as it is only by a thorough knowledge of the subject that success can be ensured.

**A Valuable Work of Reference for Practising Midwives.**

**A PRACTICAL HANDBOOK OF MIDWIFERY.**

By FRANCIS W. HAULTAIN, M.D. New and Revised Edition, profusely Illustrated with Original Cuts, Tables, &c.; handsomely bound in leather. **6/-** net, **6/3** post free

### Useful Handbooks.

**HOW TO BECOME A CERTIFIED MIDWIFE.**

By E. L. C. APPEL, M.B., B.S., B.Sc. Lond. Cloth, **1/-** net, **1/1** post free.

**NURSING NOTES ON MIDWIFERY AND GYNÆCOLOGY.**

By Sister ROSS, Rotunda Hospital, Dublin. (Suitable size for apron pocket) Cloth, **2/-** net, **2/1** post free

**THE MIDWIVES' POCKET BOOK** (With Prefatory Chapter on the Midwives Bill).

By HONNOR MORTEN. Cloth, **1/-** post free.

**THE MOTHER'S HELP AND GUIDE TO THE DOMESTIC MANAGEMENT OF HER CHILDREN.**

By P. MURRAY BRAIDWOOD, M.D. Second Edition, with Addenda, cloth, **2/-** post free.

**THE NURSING OF SICK CHILDREN.**

By JAMES BURNET, M.A., M.D., M.R.C.P.Edin. Cloth, **1/-** net, **1/1** post free.

*Contains Chapters on:—*

- The Feeding of Sick Children.
- Baths: When and How to Give Them.
- On the Management of Respiratory Cases.
- Vomiting and Diarrhœa Cases.
- On the Management of Rheumatic Cases.
- Diseases of the Nervous System.
- Paralysed and Spinal Cases.
- Rickets and Scorbutus.
- Skin Cases.
- Diseases of the Eye, Ear, Nose, and Throat.

**The Scientific Press, Ltd.,**

*Catalogue of Nursing Manuals sent post free on application.*

**Publishers of Nursing Text-Books, Charts, and Case-Books of all Descriptions.**

**28 & 29 SOUTHAMPTON STREET, STRAND, LONDON, W.C.**

